# Volumetric Scalability of Microfluidic and Semi-Batch Silk Nanoprecipitation Methods

**DOI:** 10.3390/molecules27072368

**Published:** 2022-04-06

**Authors:** Saphia A. L. Matthew, Refaya Rezwan, Yvonne Perrie, F. Philipp Seib

**Affiliations:** 1 Strathclyde Institute of Pharmacy and Biomedical Sciences, University of Strathclyde, 161 Cathedral Street, Glasgow G4 0RE, UK; saphia.matthew@strath.ac.uk (S.A.L.M.); yvonne.perrie@strath.ac.uk (Y.P.); 2 Department of Pharmacy, State University of Bangladesh, Dhaka 1205, Bangladesh; rezwan.refaya@yahoo.com; 3 School of Clinical Sciences, Faculty of Medicine, Nursing and Health Sciences, Monash University, Clayton, VIC 3168, Australia; 4 EPSRC Future Manufacturing Research Hub for Continuous Manufacturing and Advanced Crystallisation (CMAC), University of Strathclyde, Technology and Innovation Centre, 99 George Street, Glasgow G1 1RD, UK

**Keywords:** silk fibroin, nanoprecipitation, semi-batch, microfluidics, scalable manufacture

## Abstract

Silk fibroin nanoprecipitation by organic desolvation in semi-batch and microfluidic formats provides promising bottom-up routes for manufacturing narrow polydispersity, spherical silk nanoparticles. The translation of silk nanoparticle production to pilot, clinical, and industrial scales can be aided through insight into the property drifts incited by nanoprecipitation scale-up and the identification of critical process parameters to maintain throughout scaling. Here, we report the reproducibility of silk nanoprecipitation on volumetric scale-up in low-shear, semi-batch systems and estimate the reproducibility of chip parallelization for volumetric scale-up in a high shear, staggered herringbone micromixer. We showed that silk precursor feeds processed in an unstirred semi-batch system (mixing time > 120 s) displayed significant changes in the nanoparticle physicochemical and crystalline properties following a 12-fold increase in volumetric scale between 1.8 and 21.9 mL while the physicochemical properties stayed constant following a further 6-fold increase in scale to 138 mL. The nanoparticle physicochemical properties showed greater reproducibility after a 6-fold volumetric scale-up when using lower mixing times of greater similarity (8.4 s and 29.4 s) with active stirring at 400 rpm, indicating that the bulk mixing time and average shear rate should be maintained during volumetric scale-up. Conversely, microfluidic manufacture showed high between-batch repeatability and between-chip reproducibility across four participants and microfluidic chips, thereby strengthening chip parallelization as a production strategy for silk nanoparticles at pilot, clinical, and industrial scales.

## 1. Introduction

Achieving reproducibility of the critical quality attributes of nanoparticles, including the physicochemical properties, remains a significant challenge during the scale-up of nanomedicine production from the bench to the manufacturing sector [1,2,3,4]. The physicochemical properties of nanoparticles affect many performance factors, including drug loading, stability on storage [5], and in vivo performance [6]. Favorable attributes for drug carriers include nanoparticle sizes between 100 and 200 nm and a hydrophilic and negatively charged shell [7].

One promising material for fabrication of nanoparticle drug carriers is reconstituted *Bombyx mori* silk fibroin, a polymer with a robust clinical track record and demonstrated biocompatibility in vitro and biodegradability ex vivo [8,9,10,11]. The block copolymer structure of silk fibroin, hereafter referred to as silk, allows it to assume multiple polymorphic states, ranging from the amorphous, water-soluble silk I state to the highly crystalline, insoluble silk II state [11]. In the silk I state, the primary self-assembly of the hydrated, extended silk molecules into kinetically locked nanoparticles can occur under shear-induced [12] or antisolvent-induced [13,14,15] desolvation. Both mechanisms involve the breakage of the intermolecular hydrogen bonds between water molecules and the hydrophilic blocks of the silk polymer [12]. The system can be stabilized by the reorganization of the biopolymer tertiary structure to establish hydrophobic interactions and β-sheet crystal structures within and between silk molecules [12].

The micelle-like hydrophobic core and anionic hydrophilic shell structure [16,17,18] of *Bombyx mori* silk nanoparticles impart hemocompatibility ex vivo [9] and enable post-synthetic, electrostatic drug loading [13,19,20,21]. The polar amino acids, such as tyrosine, serine, histidine, and lysine, that are present on the nanoparticle surface are also amenable to covalent modification [9]. This has enabled the conjugation of cell-targeting agents (e.g., folate [22]) and the implementation of stealth technology, such as PEGylation [23], to suppress various clinically undesirable processes, such as agglomeration in physiological media [9], blood platelet coagulation ex vivo [9,24], and proinflammatory responses in vitro [9,23].

Silk nanoprecipitation in a semi-batch format is a simple, one-step procedure that involves the drop-by-drop addition of an aqueous silk solution, hereafter referred to as the silk precursor, into a water-miscible organic solvent [13,20,24]. The solvent must be present in at least 200% *v*/*v* excess and must be a poor solvent for at least one of the hydrophobic polymer blocks [25,26]. The physicochemical properties of silk nanoparticles fabricated using semi-batch nanoprecipitation have shown a dependence on the concentration and average molecular weight of the silk [15,27] and on the composition of the dissolved solute and antisolvent mixture [13,20,24,28,29]. Variations in the feed height and feed rate that can occur during the manual feeding of the silk precursor to the organic antisolvent has led to the adoption of semi-automated fluid handling systems to reduce the impact of these confounders [30]. For example, nanoparticles formed using the optimized formulation variables of a 5:1 *v*/*v* isopropanol:silk precursor [13,15], a constant flow rate of 1 mL min^−1,^ and a constant feed height of 7.5 cm showed sizes (104–134 nm) and yields (9–23%) with narrow polydispersity indices (0.12–0.14) that depended on the stirring rate [30].

The scale-up of manufacturing processes for in vivo clinical trials require changes in the production methodology by increasing the total volume of manufacture (volumetric scale-up) or by increasing the concentration of precursor (concentration scale-up) [4]. Scale-up of fabrication techniques from the bench [31] can be further aided by computational simulations to establish appropriate flow and mixing regimes [26,32]. However, changes in the critical process parameters, such as tank geometry and mixing time [3], can lead to aberrant physicochemical characteristics of the nanoparticles and potentially inaccurate safety profiling [1,4]. For example, in 2016, unreliable scale-up methods implemented with generic Doxil^®^ formulations led to reduced drug efficacy [1,33]. Investigating the reliability of silk nanoprecipitation outcomes across varying scales is, therefore, important to correct for changes occurring in the physicochemical properties during the transition from lab to pilot scales. However, the scalability of silk nanoprecipitation in a semi-batch format is poorly understood.

Continuous techniques have been developed and optimized [14,15,34] to increase the ease of volumetric scale-up [1,4]. One effective approach includes microfluidic-assisted nanoprecipitation [1,14,15]; microfluidics has emerged within the last decade as an alternative bottom-up route for silk nanoparticle manufacture. Unlike batch and semi-batch processes, the addition of a silk precursor to the antisolvent is not a time-dependent process; therefore, increased control over the mixture composition is attained in the microfluidic mixing channel compared to bulk mixing. Additionally, the semi-batch processes are influenced by macro-, meso-, and micro-mixing times, whereas microfluidic-assisted nanoprecipitation is controlled only by the micro-mixing time. Common microfluidic devices used for nanoprecipitation operate under laminar flow, in which mixing occurs by diffusion across the fluid interface and mixing times are dictated by the channel dimensions [35]. To date, microfluidics has mostly been used to form silk water-in-oil emulsions. Silk emulsions of relatively high monodispersity can be achieved using flow-focusing droplet microfluidics (145–200 µm) [36] and single T-junction droplet nanofluidics (51–1500 nm) [34]. However, the production throughput of silk water-in-oil microfluidics can be limited by the requirement for low total flow rates.

The staggered herringbone micromixer has been employed to address the need for high-throughput continuous manufacture of narrow polydispersity index nanoparticles (110–310 nm) by silk precursor desolvation [14]. The mixing efficiency of the aqueous silk precursor and antisolvent layers within the microchannel is high compared to conventional microfluidic designs [37,38] and semi-batch bulk mixing [39] due to the chaotic advection caused by the presence of the 3D asymmetric bas-relief herringbone structures [39]. Use of this micromixer for nanoprecipitation showed that the physicochemical properties of the resulting nanoparticles depended on the silk molecular weight range [15], organic antisolvent properties [14], and the composition of the aqueous and organic mixture [14]. A flow ratio of 5:1 isopropanol to silk precursor and a total flow rate of 1 mL min^−1^ provided the optimal formulation and process parameters for nanoparticle manufacture at the lab scale in batch mode and resulted in spherical, colloidally stable nanoparticles with an average size of 110 nm, average polydispersity index of 0.14, and average zeta potential of −29.8 mV [14]. The volumetric scale-up of production is achieved by increasing the microchannel diameters or by chip parallelization, although significant changes to the micromixer geometry and maximum flow rate limits can introduce scaling challenges. Consequently, investigating the scalability of the semi-batch manufacture of silk nanoparticles remains a topic of interest.

The aim of the present study was to examine the impact of the volumetric scale-up of silk nanoprecipitation on nanoparticle critical quality attributes under semi-batch and microfluidic mixing regimes using optimized formulation and process parameters [13,14,15]. With the semi-batch format, we investigated the impact of the total volume of the silk precursor and antisolvent mixture (volumetric scale) on the critical quality attributes (e.g., particle size, polydispersity, zeta potential, stability, secondary structure, morphology, and yield) of the resulting silk nanoparticles. The effect of using similar mixing times and average shear rates of mixing on nanoparticle properties during scale-up were then assessed. We used the staggered herringbone micromixer in the benchtop NanoAssemblr^TM^ platform, hereafter referred to as microfluidic format, as a scale-independent control and estimated the impact of chip parallelization on the physicochemical properties through a round robin test. To identify the bottlenecks in each format of clinical and industrial production, the throughput of both formats was estimated.

## 2. Results

### 2.1. Silk Nanoparticle Physicochemical Characterization

#### 2.1.1. Volumetric Scale-Up in Semi-Batch Format

**Figure 1:**
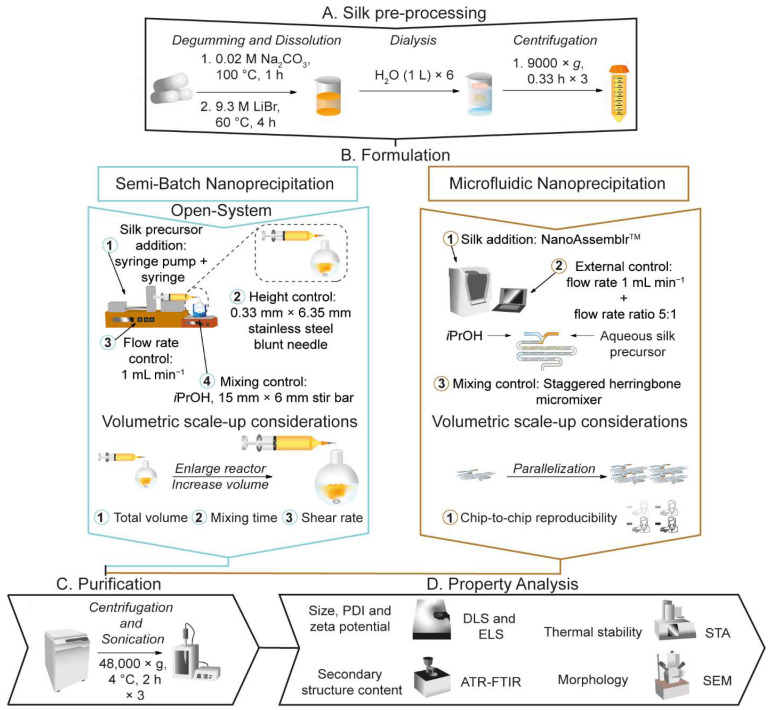
The nanoprecipitation workflow for the manufacture of silk nanoparticles in semi-batch and microfluidic formats by antisolvent-induced desolvation in isopropanol and the volumetric scale-up considerations explored in this study. The formulation process consisted of four steps in semi-batch format: (1) preparation of an aqueous silk precursor solution in a syringe equipped with a blunt needle, ensuring the silk precursor is free of bubbles; (2) fixing the needle to standardize the feed position of the silk precursor; (3) addition of the silk precursor to the antisolvent at a fixed flow rate; and (4) control of the stirring rate and mixing time during addition of the silk precursor to the antisolvent. The formulation process consisted of three steps in microfluidic format: (1) preparation of aqueous silk precursor solution and antisolvent syringes, ensuring the syringes are free of bubbles for loading into a NanoAssemblr^TM^ microfluidic chip; (2) remote control of the flow rate ratio of isopropanol:silk precursor; and (3) remote control of the mixing time via the total flow rate.

**Figure 2:**
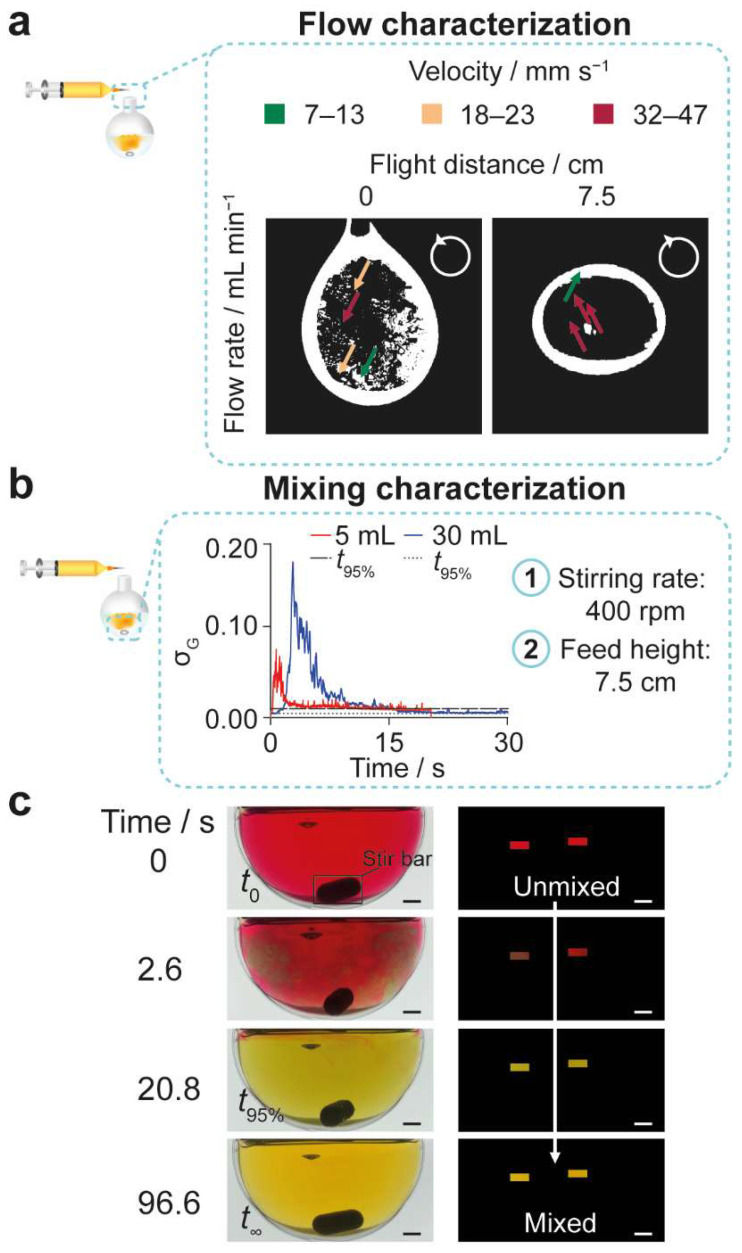
Exemplary characterization of the silk precursor droplet flow rate and the bulk mixing time in the open semi-batch system. (**a**) The droplets of silk precursor, spiked with iron oxide nanoparticles, showed circulatory flow when extruded from the 0.33-mm blunt needle at a flow rate of 1 mL min^−1^. The processed binary images showed the circulatory flow field of silk precursor droplets at free-fall heights of 0 and 7.5 cm. Insets show the direction of flow in two dimensions. (**b**) The mixing time increased as the volume of antisolvent increased and was measured using the color change of a mixture of 4.3 × 10^−3^ mg L^−1^ methyl red and 4.3 × 10^−3^ mg L^−1^ thymol blue from acidic pH (red) to neutral pH (yellow). The variation in the standard deviation of the normalized green channel (σ_G_) across the lower limits of the 6- and 36-mL volumetric scales in the open semi-batch system. (**c**) The raw and processed images showing the red to yellow color change observed using the DISMT method at the stirring rate of 400 rpm, feed height of 7.5 cm, and initial antisolvent volume of 30 mL. Scale bars = 5 mm.

In the semi-batch open system (Figure 1 and Figure 2), the impact of the volumetric scale of nanoprecipitation on the physicochemical properties of silk nanoparticles was determined in the presence or absence of stirring.

To aid the translation from lab- to pilot-scale equipment, the flow properties of the semi-batch system were first characterized (Figure 2, Tables S1–S3, Supplementary Materials). The silk molecules within the free-falling droplets were mixed mainly by convection, due to the high fluid velocities compared to the diffusion coefficient (Figure 2a, Table S3). Consequently, the silk concentration within the droplets and the solvent-antisolvent mixing efficiency across the surface of the droplets could be considered as uniform.

The mixing time and shear parameters of the reactors for the semi-batch system were then characterized. The bulk mixing time of the reactors at the 6-mL and 36-mL volumetric scales were estimated using an adapted dual indicator system for the mixing time [40,41,42] method at the initial antisolvent volumes of 5 and 30 mL, respectively. As the mixing times for the unstirred semi-batch reactors were greater than 120 s, the reactors were also stirred at 400 rpm during the addition of the silk precursor. The similarity of the mixing times at the volumetric scales of 6 (8.4 s) and 36 mL (29.4 s) was increased by stirring at 400 rpm (Table 1, Figure 2b). The shear parameters, including the power per volume, integrated shear factor, and energy dissipation, associated with stirring at 400 rpm were estimated to describe the speed of mixing for comparison with other reactor geometries and impeller types (Table S2). Although the estimated impeller tip speed of 1.97 ms^−1^ at the 400 rpm stirring rate did not change, the average shear rates decreased from 113,442–155,177 s^−1^ to 18,907–25,863 s^−1^ as the volumetric scale increased from 6 to 36 mL (Table S2).

**Figure 3:**
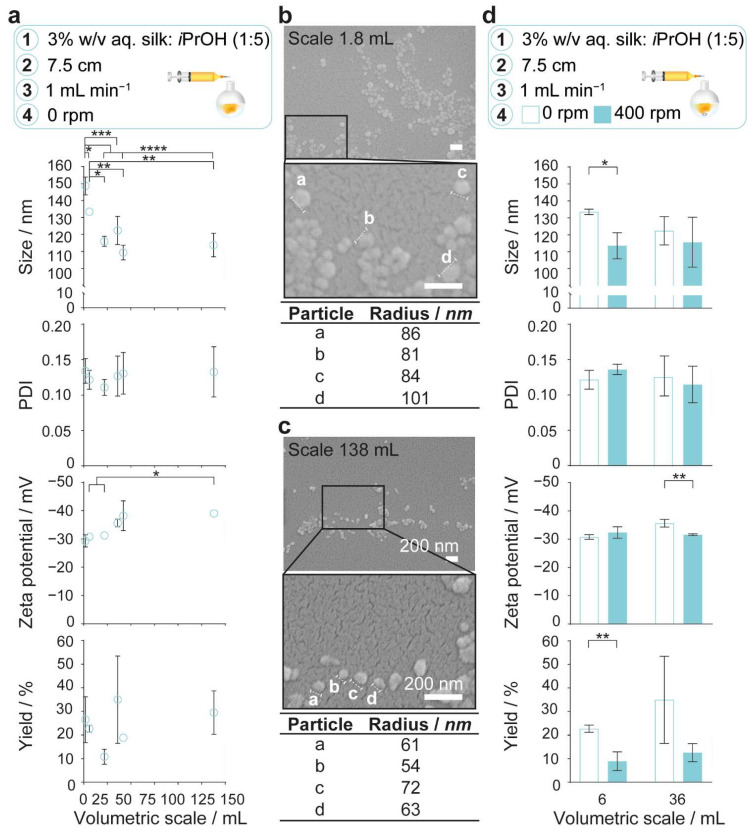
The impact of semi-batch volumetric scale on physicochemical properties and yield of silk nanoparticles. (**a**) Increasing the volumetric scale of silk desolvation in semi-batch format without stirring decreased the nanoparticle size and significantly increased the zeta potential. Multiple groups were evaluated by one-way ANOVA, followed by Tukey’s multiple comparison post hoc test. Scanning electron microscopy of the spherical nanoparticles manufactured at (**b**) 1.8-mL and (**c**) 138-mL scales. (**d**) Impact of the stirring rate and volumetric scale on nanoparticle size and yield. Two groups were evaluated by Student’s *t*-test. Data obtained from semi-batch manufacture at 6-mL scale and at 36-mL scale at the stirring rate of 400 rpm were from previously published work [30] and were included to simplify the comparison. Error bars are hidden in the plot symbols when not visible, ± SD, *n* = 3. Asterisks denote statistical significance determined using post hoc tests as follows: * *p* < 0.05, ** *p* < 0.01, *** *p* <0.001, and **** *p* < 0.0001. Scale bars = 200 nm.

In the absence of stirring, an increase in volumetric scale from 1.8 to 21.9 mL significantly decreased the silk nanoparticle size from 149 nm to 116 nm (Figure 3a). No further reduction in size occurred with further increases in the volumetric scale from 21.9 to 138 mL. The zeta potential significantly decreased from −31 to −39 mV as the scale increased from 6 to 138 mL (Figure 3a). However, the polydispersity index (0.09–0.16), yield (18–49%), and spherical morphology were not significantly affected by volumetric scale changes (Figure 3a–c). Increasing the stirring rate from 0 to 400 rpm at the 6-mL scale significantly reduced the nanoparticle size from 134 nm to 114 nm and the yield from 23% to 9% (Figure 3d). In contrast to the 6-mL scale result, stirring had no similar impact when the volumetric scale was increased to 36 mL (Figure 3d).

#### 2.1.2. Volumetric Scale-Up by Parallelization in Microfluidic Format

**Figure 4:**
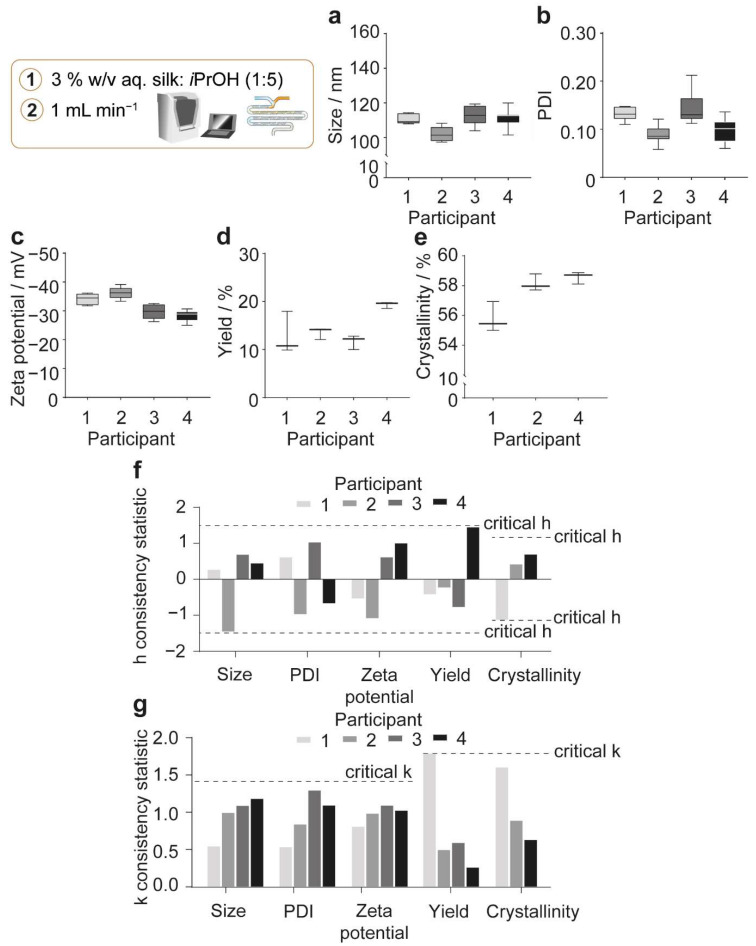
Variation observed in physicochemical properties and yield of silk nanoparticles manufactured in the NanoAssemblr^TM^ as part of a round robin study. (**a**) Hydrodynamic diameter, (**b**) polydispersity index (PDI), (**c**) zeta potential, (**d**) yield, and (**e**) β-sheet crystallinity of silk nanoparticles. (**f**) The h and (**g**) k consistency statistics of nanoparticles manufactured using four participants for all properties except for crystallinity, which were calculated using the data from three participants. Participants 2 and 4 produced four nanoparticle batches for DLS and ELS studies, and participants 1 and 3 produced three nanoparticle batches. Yield and crystallinity were analyzed across three nanoparticle batches for all participants. Data from participants 2 [43], 3 [14], and 4 [15] were published previously.

Unlike the semi-batch format, nanoprecipitation using the microfluidic format can be run at increasing volumetric scales by parallelizing the microfluidic chips without changing their internal dimensions. Therefore, the microfluidic format provided a volumetric scale-independent process. For this reason, we used the optimum formulation and process parameters in a round robin test at the 6-mL total volume of silk precursor and antisolvent (volumetric scale) to estimate the operating boundaries upon parallelization. Four participants, each equipped with a distinct microfluidic chip, were employed in the study and each participant repeated manufacture a minimum of three times using three silk precursors. The average of each nanoparticle property obtained in the microfluidic format was estimated using the grand average of all participants and gave an average nanoparticle size of 109 nm, polydispersity index of 0.11, zeta potential of −32 mV, and yield of 14% (Figure 4).

The between-participant and within-participant consistencies were characterized by the h and k statistics, respectively (Figure 4f–g). The h statistic is defined as the ratio of the difference between one participant’s average from the grand average to the standard deviation of the differences observed for all participants. The k statistic is defined as the ratio of the standard deviation for the average of one participant to the pooled standard deviation of all participants. Therefore, the h statistic indicates how comparable the averages from each participant are. Similarly, the k statistic indicates how comparable the standard deviation of the averages from each participant are. Participants that give greater variability than that of all participants are shown when the k value exceeds 1. The upper critical h and k statistics were calculated to identify outliers, by indicating whether the averages and standard deviations of each participant were sufficiently different from the others. The critical h statistic is predefined by the number of participants and replicates in the study and was calculated using equations derived from an unpaired *t*-test at the 0.5% significance level. The critical k statistic is also predefined by the number of participants and replicates in the study and was calculated using the *F* distribution at the 0.5% significance level. No outliers were observed for the physicochemical properties, yield, or crystallinity measurements, as the data did not exceed the critical h and k values (Figure 4f–g).

The probable differences between nanoprecipitation results for one operator using one microfluidic chip were estimated using the 95% limit of the repeatability precision statistic. This precision statistic was calculated using the pooled standard deviation of all participants and provides a guideline for expected differences between replicates at the 95% probability level. Similarly, the 95% limit of the reproducibility precision statistic was calculated as a guideline for the differences expected to occur, with 95% probability, between nanoprecipitation results obtained using multiple microfluidic chips and operators. This precision statistic was calculated using the sum of the pooled standard deviation of all participants (within-participant component) and a between-participant standard deviation. The between-participant standard deviation was calculated using the standard deviation of the differences observed between the averages of the participants and the grand average, while accounting for the pooled standard deviation of all participants.

The 95% repeatability precision limits of nanoparticle properties indicated that, for one microfluidic chip and operator, the maximum absolute differences in nanoparticle size, polydispersity index, zeta potential, and yield of 13 nm, 0.06, 5 mV, and 7%, respectively, between multiple nanoprecipitation replicates would be expected to occur with a 95% probability (Table S4). Expectedly, the 95% reproducibility precision limits for multiple operators and microfluidic chips were greater than the 95% repeatability limits for one operator and microfluidic chip. The 95% reproducibility limits indicated that the maximum absolute differences of 17 nm, 0.09, 11 mV, and 11%, respectively, would be expected to occur with a 95% probability between multiple nanoprecipitation repeats using different microfluidic chips (Table S4).

### 2.2. Secondary Structure Measurement

**Figure 5:**
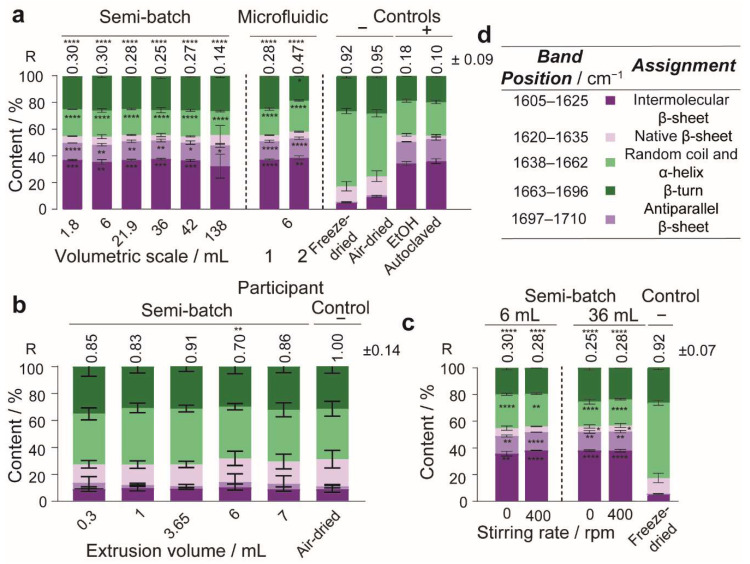
The total β-sheet content of silk nanoparticles did not vary significantly with volumetric scale when prepared by semi-batch format. FTIR secondary structure content (%) of (**a**) silk nanoparticles manufactured using different volumetric scales and formats of manufacture, (**b**) silk precursor extruded from the semi-batch open system feed needles, (**c**) silk nanoparticles manufactured using different volumetric scales and stirring rates, and (**d**) key for FTIR band assignments. The relative area of the assigned peaks in the second derivative spectrum were used to calculate the secondary structure content (%). The positive silk II controls were defined as the 70% *v*/*v* ethanol/ultrapure H_2_O annealed and the autoclaved silk films. The negative silk II controls were defined as the air-dried silk film and freeze-dried silk powder. The negative silk II control (air-dried film) was defined as the reference for the spectral correlation coefficients of all sample types. For nanoparticles manufactured at different volumetric scales, the total β-sheet, β-turn, α-helix, and random coil contents and the correlation coefficients were evaluated using the one-way ANOVA tests against the negative silk II control (freeze-dried powder) followed by the Dunnett T3 multiple comparison post hoc test. For the microfluidic format, the nanoparticle correlation coefficients, total β-sheet, β-turn, α-helix, random coil, and anti-parallel β-sheet contents were evaluated by one-way ANOVA against the negative silk II control (freeze-dried powder) followed by Dunnett’s multiple comparison post hoc test. For nanoparticles manufactured at a 6-mL scale with and without stirring, the correlation coefficients, total β-sheet, and β-turn values were evaluated by one-way ANOVA against the negative silk II control (freeze-dried powder) followed by Dunnett’s multiple comparison post hoc test. For nanoparticles manufactured at a 36-mL scale with and without stirring, the correlation coefficients, α-helix, and random coil contents and β-turn and native β-sheet values were evaluated by one-way ANOVA against the negative silk II control (freeze-dried powder) followed by Dunnett’s multiple comparison post hoc test. The remaining contents were evaluated using the Brown–Forsythe and Welch ANOVA tests. Data (±SD, *n* = 3) obtained from semi-batch manufacture at the 6-mL scale and the stirred 36-mL scale [30], silk II controls [30], and from participant 2 in the microfluidic format [43] were published elsewhere. Multiple groups of a silk precursor extruded with varying volume (±SD, *n* = 9) were evaluated by the Brown–Forsythe and Welch ANOVA against the negative silk II control (air-dried film) followed by the Dunnett T3 multiple comparison post hoc test. Asterisks denote statistical significance determined using post hoc tests as follows: * *p* < 0.05, ** *p* < 0.01, *** *p* < 0.001, and **** *p* < 0.0001.

Deconvolution of the attenuated total reflectance-FTIR (ATR-FTIR) spectra in the amide I region (1600–1700 cm^−1^) was used to assess the secondary structure content of silk nanoparticles formulated at different volumetric scales. As the critical shear rate of the 3% regenerated silk precursor [44] was exceeded in the semi-batch feed needle (Table 2), the impact of the volume of silk precursor extruded through the feed needle on shear-induced assembly was also assessed. In conjunction with spectral deconvolution, the spectral correlation coefficient method [45] was employed to estimate the overall change in the silk secondary structure incurred by the treatment of the silk precursor into processed silk samples, such as silk nanoparticles. The correlation coefficient calculation directly compared the spectra of processed silk samples against a reference silk II negative control (air-dried silk film), which was used in this study as a model for the silk precursor. Therefore, the correlation coefficient (R) indicates the similarity between the secondary structure content of processed samples and the silk precursor, where the correlation coefficient value of 1 is the maximum similarity that can be achieved. For example, the correlation coefficients of the silk II negative controls, the air-dried films (0.92–1.0), and the freeze-dried powders (0.95) demonstrated high similarity to the silk precursor (Figure 5). Conversely, the correlation coefficients of the silk II positive controls, the 70% *v*/*v* ethanol/ultrapure H_2_O annealed silk films (0.18), and the autoclaved silk films (0.10) demonstrated low similarity to the silk precursor (Figure 5a). Correlation coefficient values that lay between the boundaries of the silk II negative and silk II positive controls would be expected to exhibit an intermediate secondary structure content.

In the open semi-batch system, the silk nanoparticle β-sheet content ranged from 54–56% (Figure 5a). The crystallinity of nanoparticles manufactured at all volumetric scales in the semi-batch format compared well with those obtained using microfluidic nanoprecipitation. For example, in the microfluidic format, the grand average crystallinity of three participants was 58%, with 95% repeatability and reproducibility precision limits of 7% and 11%, respectively (Figure 4e, Table S4). The nanoparticle total β-sheet crystallinity did not depend on the volumetric scale of semi-batch nanoprecipitation (Figure 5a) and the silk precursor volume did not cause any general variation in the β-sheet content of liquid silk extruded from the feed needle, which ranged from 26–32% (Figure 5b). Additionally, the correlation coefficients, which ranged from 0.70–0.86, did not vary with the extruded silk precursor volume and reinforced the low β-sheet content measured using spectral deconvolution (Figure 5b). Similarly, the silk nanoparticle correlation coefficient did not significantly change for nanoparticles produced at the 1.8- to 138-mL volumetric scale and ranged between 0.14–0.30 (Figure 5a). The correlation coefficients at all volumetric scales compared favorably with the correlation coefficients of 0.28–0.47 obtained using the microfluidic format and provided further evidence of high β-sheet crystallinity. Nevertheless, the anti-parallel β-sheet content increased (13–16%) significantly and the intermolecular β-sheet content decreased (37–33%) significantly from the 1.8-mL to 138-mL volumetric scale. Finally, increasing the stirring rate from 0 to 400 rpm at both volumetric scales of 6 and 36 mL did not significantly affect the nanoparticle β-sheet content or the correlation coefficients (Figure 5c). The nanoparticle secondary structure did not significantly change with the stirring rate at the 36-mL scale. However, by increasing the stirring rate from 0 to 400 rpm at the 6-mL scale, the intermolecular β-sheet (36% to 38%) and anti-parallel β-sheet (13% to 14%) contents increased while the α-helix and random coil content (20% to 19%) decreased.

### 2.3. Thermal Analysis

**Figure 6:**
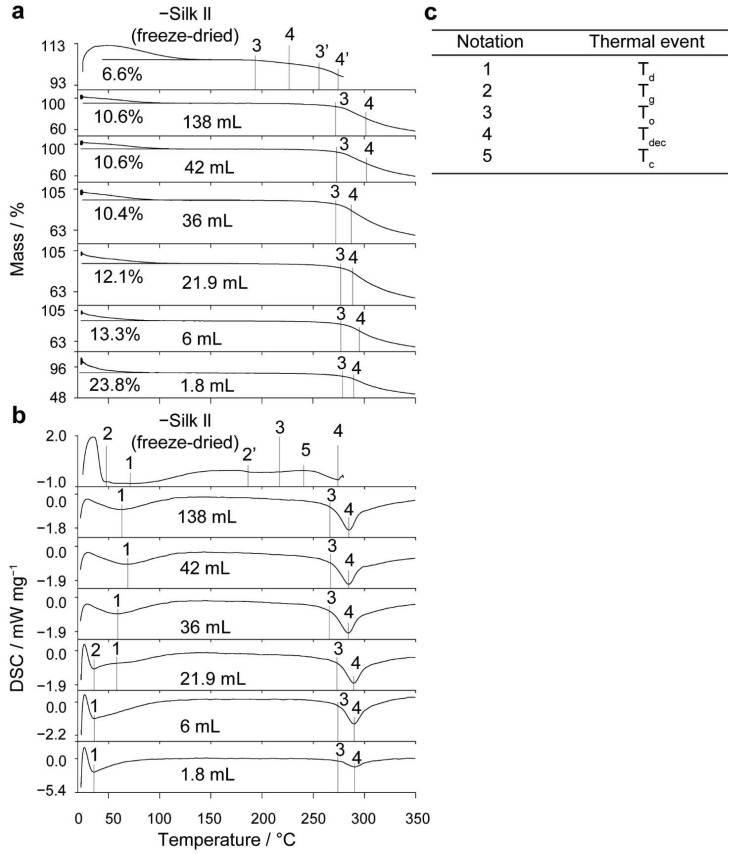
Representative first-cycle raw (**a**) TGA and (**b**) DSC thermograms of silk nanoparticles manufactured in open semi-batch format at different volumetric scales. (**c**) Thermal event assignment key. Water content (% (*w*/*w*)), dehydration temperature (T_d_), glass transition temperatures (T_g_), extrapolated onset temperature of crystallization and decomposition (T_o_), crystallization temperature (T_c_), and decomposition temperatures (T_dec_) are reported. The data shown for the negative silk II control (freeze-dried powder) were published elsewhere [30].

Table 3 and Figure 6 show the first-cycle simultaneous thermal analysis results for the silk nanoparticles manufactured in the open semi-batch system at volumetric scales between 1.8 and 138 mL in the microfluidic format and for the negative silk II controls (freeze-dried powder). The TGA of the residual water contents and the thermal stabilities of the silk nanoparticles showed a loss of adsorbed and strongly bound water between 20 and 140 °C and silk decomposition above 170 °C. Nanoparticles manufactured at volumetric scales between 6 and 138 mL had a significantly higher water content than the negative silk II control (freeze-dried powder) (5.8% (*w*/*w*)), ranging between 10.2% and 13.0% (*w*/*w*). The nanoparticle water content decreased significantly as the volumetric scale increased. Conversely, in the microfluidic format, the 5.2% (*w*/*w*) water content was not significantly different than that of freeze-dried silk. The onset decomposition temperature of the silk nanoparticles manufactured in semi-batch format ranged between 271.5 °C and 278.1 °C and were significantly higher than that of the negative silk II control (freeze-dried powder) (261.4 °C). Similarly, the onset decomposition temperature was significantly higher (271.2 °C) for the nanoparticles produced using the microfluidic format than for the silk II control (freeze-dried powder). This result confirmed the higher content of crystalline β-sheet structures in the silk nanoparticles compared to the negative silk II control. Finally, the nanoparticle decomposition temperatures from the semi-batch (293.1–299.6 °C) and microfluidic (293.4 °C) formats were significantly higher than for the negative silk II control.

The secondary structure and thermal stability of the silk nanoparticles were also evaluated by differential scanning calorimetry (Figure 6). The temperature of desorption (39.1–67.5 °C) of nanoparticles in the semi-batch format did not vary with volumetric scale. Additionally, the desorption enthalpy (−197.5 to −207.8 J g^−1^) required to remove the adsorbed water did not vary significantly from the negative silk II control between 6- and 42-mL volumetric scales but was significantly reduced at the 138-mL scale (−191.9 J g^−1^). For the microfluidic format, while the temperature of desorption (59.9 °C) did not differ from the negative silk II control (freeze-dried powder), the desorption enthalpy (−191.8 J g^−1^) for the silk nanoparticles was significantly lower. An increase in the volumetric scale had no effect on the nanoparticle onset decomposition temperatures, which ranged from 274.0–265.7 °C and were comparable to that of the negative silk II control (freeze-dried powder). The onset decomposition temperature (264.4 °C) of silk nanoparticles from the microfluidic manufacture was also similar to the negative silk II control (freeze-dried powder). The decomposition temperatures (284.0–289.5 °C) were significantly higher than the negative silk II control (freeze-dried powder) and did not significantly change with an increasing volumetric scale. Likewise, the decomposition temperature was significantly higher for the nanoparticles produced using the microfluidic format (282.1 °C) than for the negative silk II control (freeze-dried powder). Finally, increasing the stirring rate from 0 to 400 rpm at the volumetric scale of 6 mL did not significantly impact the nanoparticle water content or thermal stability (Table S5).

### 2.4. The Impact of Volumetric Scale on the Colloidal Stability of Silk Nanoparticles

**Figure 7:**
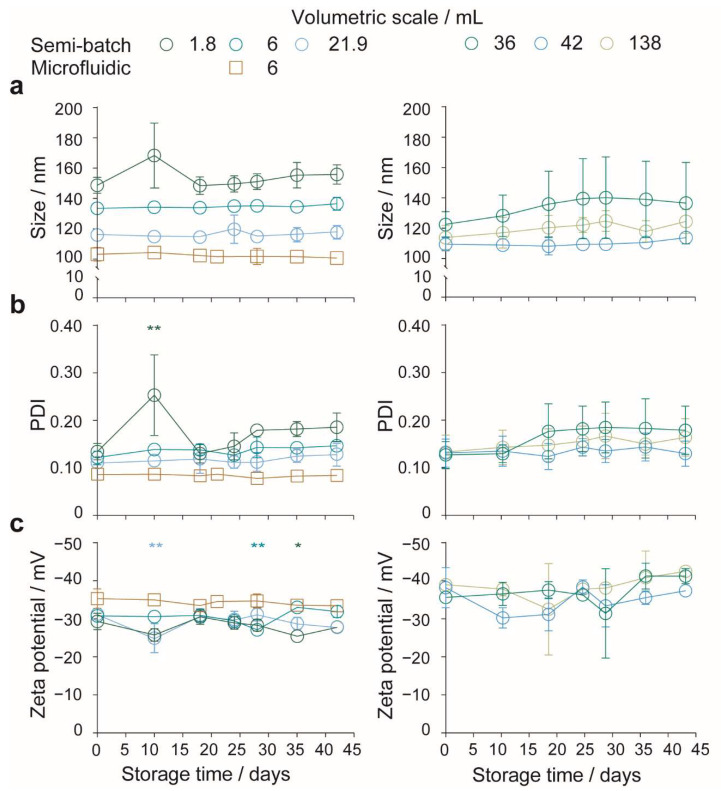
Stability of silk nanoparticles manufactured using drop-by-drop semi-batch and microfluidic formats at varying volumetric scales. (**a**) Hydrodynamic diameter, (**b**) polydispersity index (PDI), and (**c**) zeta potential of silk nanoparticles stored in water at 4 °C. Diluted nanoparticle suspensions were vortexed prior to DLS analysis, ±SD, *n* = 3. Unless otherwise stated, the silk nanoparticle stability was evaluated by one-way ANOVA followed by Dunnett’s post hoc test to compare between *t* = 0 day control and *t* > 0 day samples. The zeta potential stabilities of nanoparticles manufactured at 36- and 138-mL scale were evaluated using the Brown–Forsythe and Welch ANOVA followed by Dunnett’s T3 post hoc test. Asterisks denote statistical significance for each formulation between *t* = 0 and *t* > 0 days, determined using post hoc tests as follows: * *p* < 0.05, ** *p* < 0.01.

The short-term aqueous stability of the nanoparticles manufactured in the open semi-batch system and in a microfluidic format at volumetric scales between 1.8 and 138 mL were determined for up to 42 days by DLS and ELS (Figure 7). Nanoparticles manufactured across all volumetric scales and formats showed size stability in water for the entire duration of the study (Figure 7a); the polydispersity index and zeta potential measurements remained consistent for nanoparticles produced in a semi-batch format at volumetric scales above 21.9 mL and in a microfluidic format (Figure 7b,c). Conversely, the size polydispersity of the nanoparticles manufactured at the 1.8-mL volumetric scale in a semi-batch format increased significantly at 10 days but did not significantly differ from the initial measurement thereafter. The zeta potential of the nanoparticles manufactured at the 1.8-, 6-, and 21.9-mL volumetric scales also fluctuated across the 42 days.

## 3. Discussion

In the current study, we compared the volumetric scalability of silk nanoprecipitation in semi-batch and microfluidic formats. We used a semi-automated, open system for the semi-batch process under conditions of antisolvent-induced desolvation. The NanoAssemblr^TM^ Benchtop system equipped with a commercially available staggered herringbone micromixer, operated under conditions of shear- and antisolvent-induced desolvation, was used for the microfluidic format.

### 3.1. The Impact of Volumetric Scale on Reproducibility of Semi-Batch Silk Nanoprecipitation

The scaling issue of semi-batch nanoprecipitation is well documented [46,47]. Hence, the early identification of scaling issues for a nanoprecipitation process and the critical process parameters to maintain during volumetric scale-up can expedite the movement from lab-scale manufacture to clinical and industrial scales. However, to the best of our knowledge, this is the first example of the impact of volumetric scale and stirring rate on silk nanoprecipitation in a semi-batch format. The operation of the semi-batch open system was undertaken at a range of volumetric scales that would be suitable for pre-clinical in vitro and in vivo manufacture.

To aid translation between lab-scale and pilot-scale equipment, the flow and mixing properties of the semi-batch system were characterized. The flow rate of 1 mL min^−1^ ensured laminar flow in the syringes and needle. The maximum wall shear rate in the syringes was estimated to be between 8.9 and 261 ms^−1^, while the wall shear rate in the needle was estimated as 4724 s^−1^. Combined with the low residence time of 33 ms, these shear rates would not be expected to provide sufficient work (i.e., ≈10^5^ Pa) [48] for shear-induced nucleation of the silk molecules. In addition, the 3% *w*/*v* concentration used at all volumetric scales fell below the ≈10% *w*/*w* critical micelle concentration [49] of regenerated, aqueous silk precursors. This was confirmed by the lack of correlation between the volume of the extruded silk precursor and the β-sheet content or correlation coefficient. Consequently, the following discussion assumes homogeneous nucleation induced by desolvation and does not consider shear-induced nucleation followed by seeded crystallization.

Keeping the flow rate and silk precursor:antisolvent ratio constant but increasing the total volume resulted in a decrease in the nanoparticle size and zeta potential, although the narrow polydispersity index and yield did not change significantly (Figure 3a). Studies with polymeric and protein nanoprecipitation have indicated that the particle size decreases with the increasing mass fractions of the antisolvent [50]. In the present study, maintaining a constant silk precursor droplet volume while increasing the volume of the antisolvent caused a decrease in the initial volume ratio of silk precursor to antisolvent from 25 × 10^−3^ at a 1.8-mL volumetric scale to 0.32 × 10^−3^ at a 138-mL volumetric scale. In solvent mixtures containing a higher mass fraction of the antisolvent, the solute equilibrium concentration was reduced, thereby increasing the supersaturation along with the rate and degree of nucleation. The resulting reduction in surface-controlled nuclei growth rates with reduced local solute concentration caused a shift to a diffusion-limited growth regime. Growth was disfavored because the distance required for diffusion increased with the antisolvent volume, thereby reducing the likelihood of silk association. The resultant changes to the nanoparticle packing as the antisolvent ratio increased likely caused a greater surface exposure of acidic amino acid side chains and reduced zeta potential [51]. Finally, the Reynolds number and turbulence during droplet addition increased with the antisolvent volume; this may have decreased the micro-mixing time.

The total β-sheet content and correlation coefficients were not significantly affected by the change in supersaturation throughout volumetric scale-up. However, a significant reduction in the intermolecular β-sheet content occurred for nanoparticles manufactured at a 138-mL volumetric scale. This reflected the significantly higher anti-parallel β-sheet content in those nanoparticles than in the nanoparticles produced at smaller volumetric scales. Additionally, the volumetric scale-up caused no significant difference in desorption temperature but the water content decreased and the desorption endotherm broadened and decreased with an increasing volumetric scale (Table 3, Figure 6). This may indicate that more types of weak water-binding modes were available within the nanoparticle structure as the volumetric scale of manufacture increased. However, simultaneous thermal analysis showed no significant difference in the thermal stabilities of nanoparticles manufactured at an increasing volumetric scale (Table 3, Figure 6). This finding reinforced that the degree of β-sheet formation stayed constant [52] and indicated that the silk molecules that were incorporated into nanoparticles manufactured at a higher volumetric scale were of similar molecular weight and length distributions [15].

We speculated that increasing the similarity of mixing times and average shear rates during volumetric scale-up can increase the reproducibility of silk nanoparticle critical quality attributes. This was important for formulation screening, which is typically conducted on a small volumetric scale. For example, the physicochemical properties were made more similar with the 6-fold volumetric scale-up from 6 to 36 mL by increasing the stirring rate to 400 rpm and thereby reducing the mixing times from >120 s to 8.4 and 29.4 s, respectively (Table 1). Compared to the significant reduction in nanoparticle size with stirring at the 6-mL volumetric scale, the nanoparticle size decreased only slightly with stirring at the 36-mL volumetric scale. First, at a larger volumetric scale and in the absence of stirring, an increased degree of solvent and antisolvent mixing could occur during silk precursor addition until the critical nucleation concentration was reached, thereby enabling faster nucleation rates and the formation of smaller particles. Second, the particle size had a relatively high standard deviation for both stirred and unstirred processes. This reflected the slightly different feed point positions, which had a greater effect on the fluid dynamics and mixing times at larger volumes. Insufficient centrifugation of the smaller nanoparticles could also reduce yield. However, significant differences were still observed between the α-helix and random coil and the native β-sheet secondary structure contents of nanoparticles manufactured with stirring at the 6- and 36-mL scales. Perhaps the reproducibility of the secondary structure contents could be improved by increasing the stirring rate above 400 rpm at the 36-mL scale to further reduce the mixing time and increase the average shear rate.

### 3.2. The Reproducibility of Silk Nanoprecipitation in a Parallelizable Microfluidic Format

The NanoAssemblr^TM^ staggered herringbone micro-mixing platform provided a volumetric scale-independent platform in the microfluidic format [53]. The mixing efficiency within the microchannel depended on the flow rate and was significantly reduced at high flow rates, resulting in a high vorticity and low transverse flow (Reynolds numbers > 1000). Assuming Newtonian flow, the optimized total flow rate of 1 mL min^−1^ and the isopropanol:silk precursor flow rate ratio of 5:1 (*v*/*v*) [14,15] resulted in a Reynolds number of 40, indicating a high mixing efficiency. The complete mixing of the aqueous and organic solvents in the microchannels was likely due to the high residence time (Table 3).

Considering the wall shear rate (80,114 s^−1^) as an estimate for the maximum shear rate in the microchannel, combined with the mixing time and the residence time, the shear-induced nucleation of silk prior to the complete mixing in (and extrusion through) the microchannel exceeded both the critical shear rate [44] and critical work [48]. We speculated that the work supplied in the microchannel was sufficient for primary and secondary assembly [12] to occur in the desolvating layer prior to the complete blending of the tertiary mixture. Following the complete mixing, kinetic locking of the spherical silk nanoparticle structure was expected due to the transition from a random coil to a β-sheet secondary structure.

The microfluidic format yielded silk nanoparticles with high β-sheet crystallinity and thermal stability. The physicochemical properties of the nanoparticles were similar to those obtained using semi-batch methods with a low mixing time or high volumetric scale (e.g., ≥21.9-mL scale, stirring at 400 rpm). The low variability of the microfluidic format across batches and microfluidic chips indicated that chip parallelization with an increased total flow rate can provide a promising volumetric scale-up route. In this way, the fluid dynamics and solution composition in the micromixer are independent of volumetric scale. However, special-cause variation in micro-mixing conditions can be introduced by slight differences in the microfluidic chip dimensions. Hence, the reproducibility limit determined by the round robin study could be used as a general guide for the differences that could be expected between nanoparticles manufactured using multiple chips (Table S4, Figure 4). Importantly, silk nucleation in the feed lines could decrease the reproducibility, thereby limiting the maximum total flow rate and reducing the scalability for microfluidic manufacture. For this reason, the feed line dimensions should be selected to reduce the wall shear rate while maintaining the laminar flow. Additionally, the low nanoparticle yield could be improved by increasing the centrifugation speed and reducing the number of centrifugation cycles to sediment small nanoparticles that could not be collected at 48,400× *g*.

### 3.3. The Production Rates of Silk Nanoprecipitation in the Semi-Batch and Microfluidic Formats

The nanoparticle production rate of the semi-batch format was significantly greater than that of the microfluidic format. For example, in the semi-batch format, an intermediate nanoparticle production rate [54] of 0.53 g h^−1^ was estimated, assuming the 29% yield at the volumetric scale of 138 mL. This rate would enable 13 g of nanoparticles to be produced per day, which is suitable for pre-clinical in vitro and in vivo studies [54]. The low [54,55,56] throughput of nanoparticle production in the microfluidic format ranged from 0.040 g h^−1^ for one microfluidic chip to 0.43 g h^−1^ for 10 parallelized chips, assuming 14% yield. The resulting production of 0.96 to 10 g of nanoparticles per day would also be suitable for pre-clinical in vitro and in vivo studies but are significantly lower than the kilograms per day production rates that are required for clinical and industrial manufacture [54]. Additionally, the time and yield losses incurred by the purification process severely reduced the production rates of both formats. Consequently, increasing the centrifugation speed and reducing the centrifugation time could bring the production rates in line with industrial manufacture. In conjunction, the production throughput could be increased by raising the flow rate of the silk precursor addition to the antisolvent in the semi-batch format and by increasing the total flow rate in the microfluidic format. In both formats, increasing the flow rates can increase silk nucleation and self-assembly [14,43] and would likely require the tuning of other key formulation and process parameters, such as the silk precursor:antisolvent ratio, mixing time, and shear rate of mixing.

### 3.4. The Impact of Volumetric Scale on the Colloidal Stability of Silk Nanoparticles

A large nanoparticle surface area is beneficial for biomedical applications, but it results in a high surface energy, which can lead to a metastable nanoparticle structure. In the absence of sufficient steric and electrostatic repulsion between nanoparticles, the surface energy will be lowered by particle agglomeration and flocculation. Nanoparticles proposed for intravenous administration need to be stable under standard storage conditions to prevent clinical problems (e.g., inaccurate dosing). The characterization of the effect of aging on nanoparticle physicochemical properties is important for maximizing shelf life and preventing undesired complications. [5] For this reason, we also examined the short-term (42 days) stability [5] of silk nanoparticles manufactured in the open, semi-batch system and in the microfluidic format at 4 °C to assess storage capabilities (Figure 7).

Similar to previous studies [13,14,15], the zeta potential of nanoparticles from all formulations on the day of manufacture was lower than −25 mV, at pH ~7.4. This indicated the presence of sufficient electrostatic repulsion between particles for moderate aqueous stability. All silk nanoparticles showed size stability over the entire study period. Fluctuations in polydispersity and zeta potential occurred for nanoparticles produced from some semi-batch formulations, and, while these changes were significant, they did not follow any trend indicative of time-dependent flocculation or coagulation [14]. Dissolution also did not occur, as the size and polydispersity remained constant throughout the study.

## 4. Materials and Methods

Unless otherwise stated, the studies were conducted at 18–22 °C using reagents and solvents (>98%) obtained from Acros Organics^TM^ or Sigma Aldrich without further purification. Nanoparticle manufacture in the microfluidic format was performed in runs using the same solution of silk precursor (1 mL). Nanoparticle batches in the microfluidic format were defined as the combined product suspension from three runs using the same silk precursor solution. Independent experiments were performed in triplicate using three different silk precursor stock solutions.

### 4.1. Regeneration of B. mori Silk

*B. mori* cocoons were degummed by a 1-h treatment with sodium carbonate; aqueous solutions of the silk precursor were regenerated by dissolution in 9.3 M lithium bromide, as detailed previously [13,30].

### 4.2. General Manufacture of Silk Nanoparticles in Semi-Batch Format

An aqueous solution of 3% *w*/*v* regenerated silk precursor was added drop by drop to isopropanol in a short-neck, round-bottom flask to achieve a total volumetric ratio of 1:5 (silk:isopropanol). The silk precursor was extruded through a BD PLASTIPACK™ syringe and blunt needle (0.33 × 6.35 mm) at a constant feed rate of 1 mL min^−1^ using a syringe pump (Harvard Apparatus 22, Holliston, MA, USA) at an inclination of 0–0.1° (Figure 1). Upon the complete addition of the silk precursor, the flask was stoppered and incubated at room temperature for no longer than 2 h. Then, the mixture was diluted with ultrapure H_2_O in a polypropylene ultracentrifugation tube (43-mL capacity) and centrifuged at 48,400× *g* at 4 °C for 2 h (Beckmann Coulter Avanti^®^ J-E equipped with JA-20 rotor). The supernatant was aspirated and the pellet was resuspended in ultrapure H_2_O (20 mL), sonicated twice for 30 s at 30% amplitude with a Sonoplus HD 2070 sonicator (ultrasonic homogenizer, Bandelin, Berlin, Germany), and ultrapure H_2_O (23 mL) was added to the suspension. The centrifugation, washing, and resuspension steps were repeated three times, and the final pellet was suspended in ultrapure H_2_O (2–3 mL) and stored at 4 °C until use.

The maximum shear rate of silk under flow was calculated as the wall shear rate, assuming Newtonian flow and using the literature value for dynamic viscosity (27 mPas) [49] of 3% regenerated aqueous silk precursor and the calculated density (1.02 g mL^−1^) for the 3% *w*/*v* aqueous silk precursor solution (Table 2) [49]. For simplicity, the needle and the 3-, 10-, and 50-mL syringes used in this study were approximated as straight cylinders using the internal diameters stated by the manufacturer (Table S1). The flow regime in the needle was estimated as laminar, based on the Reynolds number of 2, which was determined using the internal diameter of the needle (330 μm) (Table 2) [57]. The upper limit of the residence time was calculated using the needle length and the linear velocity (1.94 mm s^−1^) [58].

The stirred vessels were then characterized. The Reynolds number of the stirred vessel, estimated using cylindrical geometries of approximately 3066 to 2514 at the stirring rate of 400 rpm (Table S2), indicated the occurrence of turbulent flow within the vessel at 400 rpm. The power drawn by and the power per volume of the stir bar were calculated using an estimated power number for the stir bar. The power number of the stir bar was approximated by simplifying the geometry as a one-blade, flat-paddle impeller. The empirical correlation for the maximum power number in Equation (1), as reported by Kamei and Hiraoka et al. [59,60], was then used in the fully baffled condition (as R_e_ > 200).

Maximum power number = 10(number of blades^0.7^ × (blade height/blade diameter))^1.3^ (1)

The stir bar tip speed at 400 rpm was calculated as 1.97 ms^−1^, using Equation (2) [61].

Impeller tip speed = rotational speed × π × impeller diameter(2)

The integrated shear factors, using the reactor diameters at the top surface of the stir bar and at the air–liquid interface for the initial and end volumes at the 6- and 36-mL volumetric scales, were calculated using Equation (3) [61].

Integrated shear factor = (2 × π × rotational speed × impeller diameter)/(reactor diameter − impeller diameter)(3)

The average shear rate (Equation (4) [61]) was calculated for the initial and end volumes at the 6 and 36 mL volumetric scales, using the dynamic viscosity of isopropanol and the 5:1 water:isopropanol mixture, respectively [62].

Average shear rate = √(power/(dynamic viscosity × volume))(4)

The energy dissipation per unit mass (Equation (5) [61]) was calculated for the initial and end volumes at the 6 and 36 mL volumetric scales, using the density of isopropanol and the 5:1 water:isopropanol mixture, respectively [62].

Energy dissipation per unit mass = power/(density × volume)(5)

### 4.3. Volumetric Scale-Up of Semi-Batch Silk Nanoparticle Manufacture

Silk nanoparticles were manufactured in 5 mL flasks at the 1.8 mL scale, in 10 mL flasks at the 6 mL scale, in 50 mL flasks at the 21.9, 36, and 42 mL scales, and in 150 mL flasks at the 138 mL scale. The silk precursor was added from a height of 7.5 cm, measured from the bottom of the isopropanol meniscus.

### 4.4. Dual Indicator System for Mixing Time in the Semi-Batch Format

The rotational speed of 400 rpm using an egg-shaped stir bar (15 mm × 6 mm) was investigated at the 5 mL initial antisolvent volume and an initial addition height of 7.5 cm. The rotational speed and the initial addition height at the 30-mL initial antisolvent volume were fixed at 400 rpm and 7.5 cm, respectively. Surface reflections were reduced by immersing the round-bottom flask in water within a clear acrylic box (10.3 cm × 10.3 cm × 5 cm). The flask was illuminated at a constant color temperature of 5600 K at 100% brightness by an LED panel (RALENO, Seattle, WA, USA) fixed to the back of the stirring plate.

The mixing time was measured using a dual indicator system for mixing time adapted from Melton et al. [40] and Weheliye et al. [41,42]. Stock solutions of 0.095 mg mL^−1^ thymol blue and 0.135 mg mL^−1^ methyl red in ethanol were mixed and diluted to give a working solution of 4.3 × 10^−3^ mg mL^−1^ thymol blue and methyl red in 70% *v*/*v* ethanol/ultrapure H_2_O. The working solution (5 or 30 mL) was acidified with 0.5 M HCl (0.5 mL L^−1^), and the system was equilibrated for at least 10 revolutions. An equal amount of NaOH (10.5 μL of 0.15 M NaOH for 5 mL aliquots and 10.5 μL of 0.71 M NaOH for 30 mL aliquots) was added to the mixture at a controlled feed location and height by attaching a 20-μL Eppendorf Research^®^ plus pipette (Eppendorf, Hamburg, Germany) to a clamp stand. The mixing was recorded on an iPhone SE (Apple, Cupertino, CA, USA) reverse camera at a capture speed and resolution of 240 fps and 1080 p using FiLMiC Pro (FiLMiC Inc., Seattle, WA, USA), and images were extracted using FFmpeg [63]. Each condition was repeated at least four times.

Custom MATLAB (Mathworks, Natick, MA, USA) scripts were used to apply rectangular masks of 18,000 pixels to the images and to calculate the standard deviation of the normalized green channel intensity according to the method of Rodriguez et al. [64]. The standard deviation for the fully mixed solution was defined using the average of the final 10 images, and the time required to reach 95% of the fully mixed standard deviation was defined as the mixing time (*t*_95%_) (Table 1, Figure 2).

### 4.5. Semi-Batch Droplet Analysis

#### 4.5.1. Volume and Time of Flight

The average silk precursor droplet volume at the 1 mL min^−1^ flow rate was determined by capturing the number of droplets extruded over a total silk precursor volume of 1 mL using the image capture setup detailed above.

#### 4.5.2. Fluid Velocity, Droplet Diameter, and Diffusion Scales

The flow rate within droplets extruded at the 1-mL min^−1^ flow rate and at feed heights of 0 and 7.5 cm was determined for at least three droplets of a 3% *w*/*v* silk precursor solution with 0.3% *w*/*w* iron(III) oxide (a synthetic spherical particle with 99.995% < 325-mesh [~45 µm] size, >96.8% purity, 4.6 g/cm^3^ solid density, and 0.8–1.2 g/cm^3^ bulk density; Inoxia Ltd., Sweden). The droplets were imaged on an iPhone SE (Apple, Cupertino, CA, USA) reverse camera equipped with a 15× macro lens (Shenzhen Apexel Technology Co., Guangdong, Shenzhen, China) at a focal length of 1.5 cm and were illuminated at 5600 K color temperature and at 80% brightness by an LED panel (RALENO, Seattle, WA, USA) fixed behind the droplets. The droplets were recorded at a framerate of 240 fps and resolution of 1080 p using FiLMiC Pro (FiLMiC Inc., Seattle, WA, USA), and images were extracted using FFmpeg [63]. Custom MATLAB (Mathworks, Natick, MA, USA) scripts were used for grayscale conversion, contrast-limited adaptive histogram equalization, and binary image conversion based on luminance. The particle velocities were then measured using manual tracking in ImageJ v1.52n (National Institutes of Health, Bethesda, MD, USA).

Droplet diameters were measured for at least three droplets imaged on a Photron FASTCAM SA 1.1 Model 675K M1 (Photron, San Diego, CA, USA) at 3× magnification using a Photron FASTCAM Viewer (Photron, San Diego, CA, USA). The Fickian diffusion length and time scales were calculated assuming a silk diffusion coefficient of 2.45 × 10^5^ cm^2^ s^−1^ (Table S3) [65].

### 4.6. Manufacture of Silk Nanoparticles in Microfluidic Format

Silk nanoprecipitation in microfluidic format was conducted using the NanoAssemblr™ benchtop instrument version 1.5 (model number NA-1.5-16; NanoAssemblr™, Precision Nano-Systems Inc. Vancouver, BC, Canada) equipped with a cyclic olefin copolymer microfluidic cartridge (product codes: 1207 and 1151-034 Benchtop Cartridge), as described elsewhere [14]. The fluids were introduced to the 27-mm rectangular, Y-junction, staggered herringbone micromixer (79 μm × 200 μm) through two 25 mm inlet channels. The mixing channel contained a series of raised grooves (31 μm × 50 μm) and four switchback turns [66]. The isopropanol (5 mL) and 3% *w*/*v* aqueous silk precursor solution (1 mL) were introduced through the separate chamber inlets at a flow rate ratio of 5:1 and a total flow rate of 1 mL min^−1^. The mother liquor suspension was incubated in sealed tubes for no longer than 2 h before centrifugation.

Between runs, the cartridge was cleaned with three water washes, followed by a prime. The wash procedure consisted of a 1:1-flow rate ratio of ultrapure H_2_O/ultrapure H_2_O (2 mL) at a total flow rate of 4 mL min^−1^. The priming procedure used a flow rate ratio of 5:1 isopropanol/ultrapure H_2_O, a total volume of 6 mL, and a total flow rate of 1 mL min^−1^.

The optimum formulation and process parameters were used in a round robin test in the NanoAssemblr^TM^ to estimate the repeatability and reproducibility upon parallelization. The dynamic light scattering (DLS), electrophoretic light scattering (ELS) (Zetasizer Nano-ZS Malvern Instrument, Worcestershire, UK), and yield results from four intra-laboratory participants and microfluidic chips and the β-sheet crystallinity from three participants and microfluidic chips were analyzed for consistency and precision according to ASTM E 69 (Table S4) [67]. The mixing time was estimated at 21 ms, assuming Newtonian flow [49] and using the literature viscosity value (3.14 mPas) and density (0.837 g mL^−1^) values for the 5:1 *v*/*v* isopropanol/water mixture measured at 20 °C [68]. The manufacturer’s guidelines and an analytical model for a similar system published elsewhere [57] were used for the mixing time estimation by estimating the hydraulic diameter of the channel (142 μm) and the diffusion coefficient (3.5 × 10^−10^ m^2^ s^−1^) of the 5:1 isopropanol/water mixture [62] and calculating the Peclet number (4.27 × 10^11^) to achieve a coefficient of variation of <0.1 (Table 1) [38].

The flow was determined as laminar with a Reynolds number of 40. The residence time was calculated using the total fluidic volume and flow rate [58], and complete mixing was indicated by a residence time greater than the mixing time (Table 1). For simplicity, the maximum shear rate was defined as the wall shear rate, with the assumption that chaotic advection created significantly lower shear in the channel and did not significantly alter the shear at the channel walls (Table 2). The geometry of a straight rectangular channel was used for the wall shear rate calculations, with the omission of groove depth [69].

### 4.7. Yield of Silk Nanoparticles

The total mass and total volume of the produced silk nanoparticle suspension were recorded. A known volume of the suspension was then transferred to a pre-weighed microcentrifuge tube and the total mass was recorded. The suspension was frozen at −80 °C for 5 h, freeze-dried (Christ Epsilon 1-4, Martin Christ Gefriertrocknungsanlagen GmbH, Osterode, Germany) for 24 h at −10 °C and 0.14 mbar, and the dry mass was measured. The process was repeated in duplicate, and the average yield was calculated, as described previously [30]. Freeze-dried samples were stored in a vacuum desiccator at 25 °C.

### 4.8. Physicochemical Characterization of the Silk Nanoparticles and Stability in Water

The silk nanoparticle sizes and polydispersity indexes were measured by DLS, and the zeta potentials were measured by ELS. Nanoparticle suspensions were prepared by vortexing for 20 s and sonicating twice at 30% amplitude for 30 s; measurements were made in triplicate in ultrapure H_2_O at 25 °C with refractive indices of 1.33 and 1.60 used for H_2_O and protein, respectively.

The silk nanoparticles from all studies were stored at 4 °C. The particle size and zeta potential of the silk nanoparticles generated in the open, semi-batch system and the NanoAssemblr^TM^ were determined by DLS on days 0 to 42. The nanoparticles were prepared for measurement by vortexing for 20 s at *t* > 0 days.

### 4.9. Secondary Structure Measurements of Silk Nanoparticles

Fourier transform infrared spectroscopy (FTIR) on an ATR-equipped TENSOR II FTIR spectrometer (Bruker Optik GmbH, Ettlingen, Germany) was used to analyze the secondary structures of silk films, freeze-dried powders, and freeze-dried nanoparticles. Positive silk II controls consisted of autoclaved silk films and 70% *v*/*v* ethanol/ultrapure H_2_O annealed silk films, whereas negative silk II controls consisted of air-dried silk films and freeze-dried silk powder. The air-dried silk films were drop-casted at a flow rate of 1 mL min^−1^ following extrusion from a height of 3.5 cm using varying silk precursor volumes (0.3–7 mL).

FTIR measurements were recorded in the absorption mode over 400 to 4000 cm^−1^ at 4-cm^−1^ resolution for 128 scans and then corrected for atmospheric absorption using Opus (Bruker Optik GmbH, Ettlingen, Germany). The second derivatives of the background-corrected FTIR absorption spectra were calculated in OriginLab 19b^®^ (Northampton, MA, USA) and analyzed following an adapted literature protocol [70]. The second derivative was smoothed twice using a seven-point Savitzky–Golay function with a polynomial order of 2. A non-zero linear baseline was interpolated between 2–3 of the highest values in the amide I region (1600–1700 cm^−1^). Second derivative peaks were identified in the amide I region and fitted using non-linear least squares with a series of Gaussian curves. The peak area could take any value ≤ 0; and the position, width, and height of each peak were allowed to vary. The literature band assignments [71,72,73,74] were applied to designate the relative area of each band to the relative secondary structure content.

The correlation coefficients (R) of the samples were determined according to an adapted literature protocol [45] using the air-dried silk film of an aqueous silk precursor batch as a reference. The second derivative of the absorption spectrum was calculated and smoothed twice with a five-point Savitzky–Golay function and a polynomial order of 2. The silk sample and reference were compared between 1600 and 1700 cm^−1^ according to Equation (6).

R = (∑x_i_y_i_)/(√(∑x_i_^2^∑y_i_^2^)) (6)

where x_i_ and y_i_ are the derivative values of the reference and silk sample at the frequency i.

### 4.10. Thermal Analysis of Silk Nanoparticles

Freeze-dried silk nanoparticle samples (1.06–4.89 mg) were placed in aluminum pans and subjected to differential scanning calorimetry (DSC) and thermogravimetric analysis (TGA) from 20–350 °C at a scanning rate of 10 °C min^−1^ and a nitrogen flow of 50 mL min^−1^ (STA Jupiter 449, Netzsch, Gerätebau GmbH, Germany). Thermograms were analyzed using OriginLab 19b^®^ (Northampton, MA, USA). According to previous descriptions [75], the desorption enthalpy was normalized to the corrected mass.

### 4.11. Scanning Electron Microscopy of Silk Nanoparticles

A 1-mg mL^−1^ silk nanoparticle suspension (10–20 µL) was lyophilized at −10 °C and 0.14 mbar for 24 h on a silicon wafer and then sputter-coated with gold (15 nm) using a low vacuum sputter coater (Agar Scientific Ltd., Essex, UK, and ACE200, Leica Microsystems, Wetzlar, Germany). The wafer was imaged using an FE-SEM SU6600 instrument (Hitachi High Technologies, Krefeld, Germany) at 5 kV and 40-k magnification and the images were processed using ImageJ v1.52n (National Institutes of Health, Bethesda, MD, USA), Adobe Lightroom, and Abode Illustrator (Adobe, San Jose, CA, USA).

### 4.12. Statistical Analyses

Data were analyzed using Microsoft^®^ Excel^®^ 2019 (Microsoft Office 365 ProPlus Software, Redmond, WA, USA), Minitab^®^ (Minitab^®^ Statistical Software, State College, PA, USA), and GraphPad Prism 8.2.1 (GraphPad Software, La Jolla, CA, USA), and normality of the data distributions was assumed. Two groups and multiple groups were analyzed for equivalence of variance with the F-test and Bartlett’s test. Two groups were analyzed using the independent *t*-test, with Welch’s correction applied in cases of unequal variance. Multiple groups were assessed either by one-way analysis of variance (ANOVA), followed by Tukey’s pairwise multiple comparison post hoc test, or by the Brown–Forsythe and Welch ANOVA tests, followed by the Dunnett T3 pairwise multiple comparison post hoc test. Silk nanoparticle stability was evaluated by ANOVA, followed by Dunnett’s or the Dunnett T3 post hoc test, to compare between *t =* 0 day control and *t* > 0 day samples. Statistical significance was identified using post hoc tests and defined as follows: * *p* < 0.05, ** *p* < 0.01, *** *p* < 0.001, and **** *p* < 0.0001. The number of experimental repeats (*n*) are shown in each figure legend and the data are displayed as the mean ± standard deviation.

## 5. Conclusions

Progressing the production of silk nanomedicines from bench to market requires insight into the impact of the volumetric scale on the resulting physicochemical properties of the final nanoparticle product. Here, we assessed the scalability of silk nanoprecipitation in a semi-batch format and used silk nanoparticle manufacturing in a microfluidic format as a volumetric scale-independent control. We found that using homogeneous nucleation conditions without stirring (mixing time > 120 s) decreased the particle size, surface charge, intermolecular β-sheet content, anti-parallel β-sheet content, and thermal properties as the volumetric scale increased (138 mL < 1.8 mL) due to an increase in initial supersaturation and a reduction in micro-mixing times. However, the narrow polydispersity index, spherical morphology, and high total β-sheet crystallinity of the silk nanoparticle batches did not vary with volumetric scale or with manufacturing format. The nanoparticles manufactured in a semi-batch format at scales equal to and greater than 21.9 mL had similar physicochemical properties to nanoparticles manufactured in the scale-independent, microfluidic format. Additionally, nanoparticles from all formulations, when stored as aqueous suspensions, showed short-term stability over 1 month at 4 °C. Nanoparticles prepared in semi-batch format showed greater reproducibility on a 6-fold volumetric scale-up from 6 to 36 mL total volume with active stirring at 400 rpm (mixing times 8.4 s and 29.4 s, respectively). Conversely, a round robin study involving four participants and microfluidic chips showed that manufacturing operating under conditions of high shear in the staggered herringbone micromixer resulted in high between-participant and within-participant reproducibility, with no outliers observed for nanoparticle size, polydispersity index, zeta potential, yield, or crystallinity. Strategies for increasing the scalability of silk nanoparticle manufacture include maintaining similar mixing times and shear rates of bulk mixing for the semi-batch format and using chip parallelization for the microfluidic format.

## Figures and Tables

**Table 1:** Mixing characteristics of the semi-batch system with increasing total volume, measured using the DISMT method, and the estimated mixing properties in microfluidic format. As the volumetric scale increased in semi-batch format, the bulk mixing time increased while the micro-mixing time was unaffected by microfluidic chip parallelization.

Semi-Batch Format			Microfluidic Format	
Total Volume/mL	Stirring Rate/rpm	Mixing Time/s	Residence Time/ms	Mixing Time/ms
5	400	8.4 ± 4.4	120	21
30	400	29.4 ± 6.0		

**Table 2:** Estimated flow properties of the open semi-batch format and the microfluidic format ^1^. Shear-induced and antisolvent-induced nucleation of silk were probable in microfluidic format and at a large volumetric scale in the semi-batch format.

Semi-Batch Format					Microfluidic Format	
Needle Internal Diameter/mm	Flow Rate/mL min^−1^	Residence Time/ms	Maximum Shear Rate/s^−1^	Re	Maximum Shear Rate/s^−1^	Re
0.33	1.00	43	4724	2.4	80,114	40

^1^ The wall shear rate under laminar flow is reported assuming Newtonian flow. A simplified rectangular geometry was used for the micromixer by removing the groove depth. The linear velocity was used to calculate the residence times in the fluid line and the needle in the semi-batch system while the volumetric flow rate was used for the calculation of the micromixer residence time.

**Table 3:** First-cycle thermal properties of silk nanoparticles produced by varying volumetric scale.

	Thermal Property	Semi-Batch						Microfluidic	Negative Silk II Control
		Volumetric Scale/mL							
		1.8 ^1^	6	21.9	36	42	138	6	Freeze-Dried Powder
**DSC**	**T_g_**/°C	-	59.3 ± 0.01	57.9 ^1^	58.6 ± 1.0 ^2^	-	-	71.6 ^1^	47.7 ± 0.5
	**T_d_/**°C	35.7	39.1 ± 5.3	54.2 ± 15.9	43.5 ± 13.5	66.2 ± 2.8	67.5 ± 4.2	59.9 ± 0.3	60.7 ± 8.8
	**∆H_d_/**J g^−1^	−267.7	−207.8 ± 98.0	−197.5 ± 4.6	−197.5 ± 30.1	−211.8 ± 15.8	−191.9 ± 8.9	−191.8 ± 0.9	−276.9 ± 4.21
	**T_g_’/**°C	-	-	-	-	-	-	-	184.5 ± 0.7
	**T_c_/**°C	-	-	-	-	-	-	-	241.0 ± 0.8
	**∆H_c_**/J g^−1^	-	-	-	-	-	-	-	9.9 ± 2.3
	**T_o_/**°C	274.1	274.0 ± 0.3	273.4 ± 0.6	267.4 ± 1.7	267.8 ± 0.8	265.7 ± 1.5	264.4 ± 0.1	-
	**T_dec_/**°C	290.4	289.5 ± 0.5	289.5 ± 0.3	284.7 ± 0.4	284.9 ± 0.5	284.0 ± 1.0	282.1 ± 0.4	274.9 ± 1.1 ^1^
**TGA**	**Water content/**% (*w*/*w*)	23.8	13.0 ± 1.7	11.7 ± 0.8	11.2 ± 0.7	10.7 ± 0.7	10.2 ± 0.6	5.2 ± 0.5	5.8 ± 0.8
	**T_o_/**°C	278.8	277.3 ± 0.2	278.1 ± 1.0	272.3 ± 0.1	273.2 ± 0.6	271.5 ± 0.9	271.2 ± 0.5	198.5 ± 2.2
	**T_o_’/**°C	-	-	-	-	-	-	-	261.4 ± 2.0
	**T_dec_/**°C	289.7	299.6 ± 6.6	293.1 ± 4.1	299.0 ± 10.3	297.7 ± 5.5	298.1 ± 11.3	293.4 ± 5.8	222.3 ± 13.3
	**T_dec_’/**°C	-	-	-	-	-	-	-	275.0 ± 2.7

^1^ n = 1; ^2^ n = 2.

## Data Availability

All data supporting this research are openly available from https://doi.org/10.15129/898f337a-a43b-4690-8f30-c24a77297227 (accessed on 5 April 2022).

## Supplementary Materials

The following supporting information can be downloaded at https://www.mdpi.com/article/10.3390/molecules27072368/s1. Table S1: Estimated flow characteristics of the syringes used in the open semi-batch system. Table S2: Estimated flow characteristics of the reactors used in the open semi-batch system. Table S3: Droplet characteristics impacting flow and mixing-induced silk self-assembly [65]. Table S4: Participant and precision statistics of the round robin study. Table S5: First-cycle simultaneous thermal analysis data of silk nanoparticles manufactured at different stirring rates [30].

## Sample Availability

Samples of silk nanoparticles manufactured in semi-batch format and in microfluidic format by participant 1 are available from the authors.
