# Supplementary material for: Volumetric Scalability of Microfluidic and Semi-Batch Silk Nanoprecipitation Methods
Source: Molecules. 2022 Apr 6;27(7):2368. doi: 10.3390/molecules27072368 (PMC9000471; doi:10.3390/molecules27072368)
Supplement: Supplementary file 1 [file molecules-27-02368-s001.zip › molecules-1604288-Supplementary.pdf]

Supporting Information

Saphia A. L. Matthew,[†] *Refaya Rezwan,* [‡, §] *Yvonne Perrie*[†] *and F. Philipp Seib*[*, †, $]

* Corresponding author: Tel: +44 (0) 141 548 2510; E-mail: philipp.seib@strath.ac.uk.

† Strathclyde Institute of Pharmacy and Biomedical Sciences, University of Strathclyde, 161 Cathedral Street, Glasgow, G4 0RE, U.K.

‡ Department of Pharmacy, ASA University Bangladesh, 23/3 Bir Uttam A.N.M Nuruzzaman Sarak, Dhaka 1207, Bangladesh.

§ School of Clinical Sciences, Faculty of Medicine, Nursing and Health Sciences, Monash University, Clayton, Victoria 3168, Australia.

$ EPSRC Future Manufacturing Research Hub for Continuous Manufacturing and Advanced Crystallisation (CMAC), University of Strathclyde, Technology and Innovation Centre, 99 George Street, Glasgow G1 1RD, U.K.

Number of pages: S1-S4

Number of tables: S1-S5

Table S1. Estimated flow characteristics of the syringes used in the open semi-batch system.

Syringe / *mL*	Volume / *mL*	Flow rate / *mL min^{-1}*	Residence time / *s*	Critical work / *10^{-3} Pa*	Maximum shear rate / *s^{-1}*	Volumetric flow rate / *10^{-10} m^3 s*	Average velocity / *10^{-5} m s^{-1}*	Cross-sectional area / *10^{-5} m^2*	R_e
3	1	1.000	18	33.2	0.261	167	28.3	5.89	0.093
	0.3		60	111					
10	3.65	1.000	219	18.3	0.0557	1667	10.1	165	0.055
	6		360	30.1					
	7		420	35.2					
50	23	1.000	1380	2.96	0.00892	1667	2.98	56.0	0.030

Table S2. Estimated flow characteristics of the reactors used in the open semi-batch system.

Volumetric Scale / *mL*	Volume / *mL*	R_e	Power per volume[d] / *W m^{-3}*	Average shear rate / *s^{-1}*	Energy dissipation / *W kg^{-1}*	Integrated shear factor / *s^{-1}*
**6**	5	3066 [a,c]	291	155,177	0.37	494[e]
						263[f]
	6	2514 [a,b]	242	113,442	0.29	494[e]
						263[f]
**36**	30	3066 [a,c]	48	25,863	0.06	329[e]
						132[f]
	36	2514 [a,b]	40	18,907	0.05	329[e]
						141[f]

a. Determined using the frequency of 42 rps, stir bar dimensions of 15 × 6 mm.
b. Determined using the dynamic viscosity (0.0032 kg ms^{-1}) and density (837 kg m^{-3}) of the 5:1 mixture of silk precursor: isopropanol.

c. Determined using the dynamic viscosity (0.0024 kg ms^{-1}) and density (785 kg m^{-3}) of isopropanol.
d. Calculated using the maximum power number of the stir bar (3.04) and power drawn (0.00145 W).
e. Determined using the reactor diameter for 6 mL scale (23 mm) and 36 mL scale (27 mm) at the top surface of the stir bar.
f. Determined using the reactor diameter for 6 mL scale (30 mm) and 36 mL scale (43–45 mm) at the isopropanol air-liquid interface.

Table S3. Droplet characteristics impacting flow and mixing-induced silk self-assembly. ± SD, *n = 3.*

**Height / *cm***	**Flow rate / *mL min^{-1}***	**Number of drops / *mL^{-1}***	**Droplet Volume / *µL***	**Droplet diameter / *mm***	**Diffusion length scale^a/ *µm***	**Diffusion time scale^a / *s***	**Time of flight / *s***	**Droplet velocity / *m s^{-1}***	**Fluid velocity / *mm s^{-1}***
7.5	1.000	18 ± 1	56.6 ± 1.9	3.38 ± 0.10	40.1 ± 0.4	780 ± 48.0	0.110 ± 0.002	0.918 ± 0.020	26 ± 14

a. Determined using the droplet diameter, droplet time of flight and the silk diffusion coefficient of 2.45 × 10^5 cm^2 s^{-1}[65].

Table S4. Participant and precision statistics of the round robin study.

**Physicochemical property**	**$\bar{x}$**	**$s_{\bar{x}}$**	**s_r**	**s_R**	**r**	**R**
**Size / *nm***	109	4	17	6	13	17
**PDI**	0.11	0.02	0.02	0.03	0.06	0.09
**Zeta Potential / *mV***	-32	2	3	4	5	11
**Yield / *%***	14	2	3	4	2	4
**Crystallinity / *%***	58	1	1	2	7	11

Table S5. First cycle simultaneous thermal analysis data of silk nanoparticles manufactured at different stirring rates. Data has been published elsewhere[30].

	Thermal Property	Semi-batch Stirring rate / *rpm* 0	Semi-batch Stirring rate / *rpm* 400
DSC	T_g / °C	59.3 ± 0.01	59.3[a]
	T_d / °C	39.1 ± 5.3	43.6 ± 13.9
	ΔH_d / $J\ g^{-1}$	-207.8 ± 98.0	-239.4 ± 18.8
	T_g' / °C	-	206.0[a]
	T_c / °C	-	-
	ΔH_c / $J\ g^{-1}$	-	-
	T_o / °C	274.0 ± 0.3	-
	T_{dec} / °C	289.5 ± 0.5	268.9 ± 9.0
TGA	Water content / % (w/w)	13.0 ± 1.7	284.2 ± 7.7
	T_o / °C	277.3 ± 0.2	12.6 ± 2.0
	T_o' / °C	-	274.3 ± 3.8
	T_{dec} / °C	299.6 ± 6.6	-
	T_{dec}' / °C	-	304.0 ± 4.6

a. *n* = 1
